# Tradeoffs between Maize Silage Yield and Nitrate Leaching in a Mediterranean Nitrate-Vulnerable Zone under Current and Projected Climate Scenarios

**DOI:** 10.1371/journal.pone.0146360

**Published:** 2016-01-19

**Authors:** Bruno Basso, Pietro Giola, Benjamin Dumont, Massimiliano De Antoni Migliorati, Davide Cammarano, Giovanni Pruneddu, Francesco Giunta

**Affiliations:** 1 Department of Geological Sciences and W.K. Kellogg Biological Station, Michigan State University, East Lansing, MI, United States of America; 2 Sezione di Scienze Agronomiche, Coltivazioni Erbacee e Genetica (SACEG), Dipartimento di Agraria, Università di Sassari, Sassari, Italy; 3 Institute of Future Environment, Queensland University of Technology, Brisbane, QLD, Australia; 4 James Hutton Institute, Invergowrie, Dundee, DD2 5DA, Scotland, United Kingdom; Università Politecnica delle Marche, ITALY

## Abstract

Future climatic changes may have profound impacts on cropping systems and affect the agronomic and environmental sustainability of current N management practices. The objectives of this work were to i) evaluate the ability of the SALUS crop model to reproduce experimental crop yield and soil nitrate dynamics results under different N fertilizer treatments in a farmer’s field, ii) use the SALUS model to estimate the impacts of different N fertilizer treatments on NO_3_^-^ leaching under future climate scenarios generated by twenty nine different global circulation models, and iii) identify the management system that best minimizes NO_3_^-^ leaching and maximizes yield under projected future climate conditions. A field experiment (maize-triticale rotation) was conducted in a nitrate vulnerable zone on the west coast of Sardinia, Italy to evaluate N management strategies that include urea fertilization (NMIN), conventional fertilization with dairy slurry and urea (CONV), and no fertilization (N0). An ensemble of 29 global circulation models (GCM) was used to simulate different climate scenarios for two Representative Circulation Pathways (RCP6.0 and RCP8.5) and evaluate potential nitrate leaching and biomass production in this region over the next 50 years. Data collected from two growing seasons showed that the SALUS model adequately simulated both nitrate leaching and crop yield, with a relative error that ranged between 0.4% and 13%. Nitrate losses under RCP8.5 were lower than under RCP6.0 only for NMIN. Accordingly, levels of plant N uptake, N use efficiency and biomass production were higher under RCP8.5 than RCP6.0. Simulations under both RCP scenarios indicated that the NMIN treatment demonstrated both the highest biomass production and NO_3_^-^ losses. The newly proposed best management practice (BMP), developed from crop N uptake data, was identified as the optimal N fertilizer management practice since it minimized NO_3_^-^ leaching and maximized biomass production over the long term.

## Introduction

Nitrate (NO_3_^-^) leaching from agricultural land is a pervasive problem in areas with intensive agricultural production [1–4]. Application of N fertilizer in agricultural fields, while necessary to achieve adequate levels of crop production and quality, is often associated with significant environmental impacts because it is difficult to match crop N demand with N supply [5]. In areas where farmers rely on manure or slurry as a source of N, application of excessive amounts of these organic wastes can result in significant loss of nutrients and lead to environmental degradation [6]. Groundwater contamination due to NO_3_^-^ leaching has received particular consideration in European Union legislation because pollution of this valuable resource has significantly increased in areas where intensive agriculture is practiced [4]. Excessive N applications are both economically and environmentally costly [5] and lead to large N surpluses in the soil and/or N losses [6–8]. Under field conditions, N losses are mainly due to NO_3_^-^ leaching to the groundwater, ammonia volatilization from the leaves of N-rich plants, and emission of nitrogen (N_2_) and nitrous oxide (N_2_O) to the atmosphere [9–18].

Animal manure, if properly managed, provides both physical and chemical benefits to a crop system. The effect of manure application on NO_3_^-^ leaching has been quantified by many researchers with results that have been contradictory. For example, [6] reported that use of manure in maize-alfalfa rotations in the Midwest U.S. caused high levels of NO_3_^-^ leaching when compared to inorganic N fertilization. This was attributed to high NO_3_^-^ concentration flushes that occur in the spring after the soil thaws. However, [18] reported that application of slurry in a maize-oat rotation in central Portugal caused less NO_3_^-^ leaching than mineral fertilization. Similar results were reported by [19] for a maize system in northern Italy, where organic fertilization reduced NO_3_^-^ leaching between 20 to 50%. [20] also reported positive effects for manure compost on NO_3_^-^ leaching. In contrast, [21] reported no significant differences in NO_3_^-^ leaching between dairy slurry and mineral fertilizers in southern Chile.

[22] conducted a meta-analysis on 32 published studies and found a worldwide average of 22% of N fertilizer applied to wheat and 15% of N fertilizer applied to maize systems is lost as NO_3_^-^ leaching. This suggests that it is crucial to identify regional best management strategies of agricultural N application to effectively reduce NO_3_^-^ leaching losses.

When accurately verified, crop simulation models can be useful tools to evaluate the effects of various practices on crop N uptake, production, and environmental quality [23–29]. Evaluation of a given crop model is an important first step in its application. Integration of field data with crop simulation models has been shown to be crucial to the development of a precise, long-term assessment of NO_3_^-^ leaching in relation to various N fertilizer management strategies [30–34].

Projections of climate data predict several changes in future climatic conditions which include increased atmospheric CO_2_ concentrations, increased air temperatures and altered rainfall patterns [35]. Such changes will affect crop development and therefore a crop’s ability to obtain N from the soil. For example, at the middle and higher latitudes of Europe, higher temperatures are expected to reduce the duration of the crop cycle and its N uptake capacity. In addition, the rate of carbon decomposition is predicted to increase which can result in accumulation of N in the soil and thereby increase NO_3_^-^ leaching potential [36]. [8] reported that conventional management practices in this area often result in NO_3_^-^ concentrations that range from 40 mg L^-1^ (just below the 50 mg L^-1^ maximum threshold) to as much as 120 mg L^-1^. The hypothesis of this research is that N management strategies that comply with the European legislation under current climatic conditions may not be capable of offsetting additional NO_3_^-^ leaching predicted under future climatic conditions. The objectives of this work were to i) evaluate the ability of the SALUS crop model to reproduce experimental results for yield and soil N dynamics under various N fertilizer treatments, ii) estimate the impacts of various N treatments on NO_3_^-^ leaching under predicted future climate conditions, and iii) identify the N management strategy that best demonstrates the ability to minimize NO_3_^-^ leaching and maximize yield in a NVZ under predicted future Mediterranean climatic conditions.

## Materials and Methods

### Site description, field trials, and agronomic management

A field experiment was conducted during the 2010 and 2011 growing seasons on a commercial farm near Arborea (Latitude 39° 46' 26" N, Longitude 08° 34' 53" E, 7 m a.s.l.), on the west coast of Sardinia, Italy. Permission to carry out this study was granted by the owner of the farm. The field study did not involve endangered or protected species. Extensive reclamation has been done in this area since 1930 to improve the soil which has included addition of a sand layer to improve drainage properties. Over time, establishment of commercial dairy and grain farming operations in the area has led to a sharp increase in application of both organic and inorganic N to these soils. The region currently has about 35,600 dairy cattle raised in intensive systems. Additional details on the geographical and agronomic characteristics of the area are given in [8].

Three N fertilization treatments were evaluated in this study: nil N fertilization (N0), mineral N (urea) fertilization (NMIN), and organic (cattle slurry) plus mineral N (urea) (CONV), the conventional fertilization practice adopted by farmers in the area. Detailed information regarding the time of application and fertilizer rates for 2010 and 2011 are reported in Table 1. The 4-hectare experimental field was divided into three parts, one for each fertilization treatment. However, due to the irregular shape of the field trial, areas allocated to each treatment were not uniform and consisted of 1.76 ha, 2.00 ha and 0.24 ha for the NMIN, CONV, and N0 treatments, respectively.

**Table 1:** Fertilizers and organic amendments rates, N content and dates of application.

Crop	Date (mm/dd/yy)	Treatment	Fertilizers and amendments	Amount (t ha^-1^)	N (%)	kg N ha^-1^
Maize	06/10/2010	N MIN	Urea	0.20	46.00	92.0
Maize	06/24/2010	N MIN	Urea	0.20	46.00	92.0
Maize	07/07/2010	N MIN	Urea	0.10	46.00	46.0
Maize	06/10/2010	CONV	Slurry	45.00	0.37	166.5
Maize	06/24/2010	CONV	Urea	0.20	46.00	92.0
Maize	07/07/2010	CONV	Urea	0.10	46.00	46.0
Total N distributed Maize	2010	N MIN	Urea			230.0
Total N distributed Maize	2010	CONV	Slurry + Urea			304.5
Total N distributed Maize	2010	N 0	-	-	-	-
Triticale	10/03/2010	N MIN	Urea	0.15	46.00	69.0
Triticale	02/10/2011	N MIN	Urea	0.20	46.00	92.0
Triticale	10/03/2010	CONV	Slurry	43.00	0.37	159.0
Triticale	02/10/2011	CONV	Urea	0.20	46.00	92.0
Total N distributed Triticale	2010–2011	N MIN	Urea			161.0
Total N distributed Triticale	2010–2011	CONV	Slurry + Urea			251.0
Total N distributed Triticale	2010–2011	N 0	-	-	-	-
Maize	05/18/2011	N MIN	Urea	0.20	46.00	92.0
Maize	06/01/2011	N MIN	Urea	0.20	46.00	92.0
Maize	06/14/2011	N MIN	Urea	0.10	46.00	46.0
Maize	05/18/2011	CONV	Slurry	42.50	0.32	136.0
Maize	06/01/2011	CONV	Urea	0.20	46.00	92.0
Maize	06/14/2011	CONV	Urea	0.10	46.00	46.0
Total N distributed Maize	2011	N MIN	Urea			230.0
Total N distributed Maize	2011	CONV	Slurry + Urea			274.0
Total N distributed Maize	2011	N 0	-	-	-	-

Pioneer hybrid maize cultivars (PR31A34, FAO class 700 in 2010 and PR32F73, FAO class 600 in 2011) were planted at seven plants m^-2^ with an inter-row spacing of 75 cm on June 13, 2010 and May 21, 2011. The field was tilled in both growing seasons with a chisel plow to a depth of 25 cm, and the seedbed was finalized with a rotary harrow to 10 cm. The total irrigation supplied using permanent sprinkler systems in 2010 was 4140 m^3^ ha^-1^, split into 11 applications. In 2011 the total amount applied in 15 applications was 5740 m^3^ ha^-1^. Weed control was accomplished both years of the study with Syngenta LUMAX (S-metolachlor 31.25% + Terbuthylazine 18.7% + Mesotrione 3.75%). Crops were harvested for silage on September 14, 2010 and September 8, 2011.

The “Agrano” triticale cultivar was planted October 4, 2010 with a row spacing of 15 cm and a seeding rate of 200 kg ha^-1^. The field was prepared by chisel-plowing to a depth of 30 cm and the seedbed was completed with a rotary harrow to a depth of 10 cm. To avoid water stress, triticale was irrigated with 300 m^3^ ha^-1^ applied by sprinklers and split into two applications, one in October and another in December 2010. Triticale was harvested for silage on May 10, 2011.

### Climate data

Historical weather data of daily minimum and maximum temperature, solar radiation, and rainfall (1959–2013) were obtained from the nearby meteorological station located at the “Santa Lucia experimental farm” (Zeddiani, OR; latitude 39°56'03.11''N, longitude 8°41'13.41''E, 15 m a.s.l.) of the University of Sassari. Historical daily weather data were used as input for the crop simulation model to simulate crop growth with the different treatments.

Projections of future climate data were generated with DSSAT-Perturb software [37]. The altered weather data formats are compatible with the SALUS crop model used in this study. The software follows the IPCC Fifth Assessment Report and uses CMIP5 datasets with different emission scenarios. The data were processed by a pattern scaling method, and then were re-gridded to a common 720*360 grid (0.5°*0.5°) using a bilinear interpolation method. The software uses four different Representative Concentration Pathways (RCPs) which represent four greenhouse gas concentration trajectories as adopted by the IPCC Fifth Assessment Report. The four RCPs in DSSAT-Perturb were RCP2.6, RCP4.5, RCP6.0 and RCP8.5 as associated to a range of plausible radiative forcing values of 2.6, 4.5, 6.0, and 8.5 W m^-2^, respectively. The RCP6.0 and RCP8.5 scenarios were chosen for use in this study because they were identified as having the highest probability of occurrence given current emissions trends [37, 38] Simulated data from these two RCPs were compared with simulated data from a baseline scenario (BL) using local historic weather data from 1959 to 2013.

The Global Circulation Models (GCMs) data in the DSSAT-Perturb were obtained from the Earth System Grid (ESG) data portal for CMIP5. Twenty-nine GCM models were selected in order to capture the variability between GCMs (Table 2) and pattern scaling was used to process the data. This method is based on the assumption that a simple climate model will correctly characterize the global responses of a GCM (even for non-linear responses), and that a wide range of climatic variables in a given GCM are a linear function of its changes in global annual mean temperature at different spatial-temporal scales [39, 40]. More details on the methodology and the software used were reported on the CLIM systems manual (http://www.climsystems.com/dssat-perturb/).

**Table 2:** List of the CMIP5 GCMs used in this study as future projection climate data in SALUS model.

	Model	Country	Spatial resolution for atmospheric variable (longitude*latitude)	GCM source
1	ACCESS1.0	Australia	192*145	Commonwealth Scientific and Industrial Research Organization (CSIRO) and Bureau of Meteorology (BOM), Australia
2	BCC-CSM1-1-m	China	320*160	Beijing Climate Center, China Meteorological Administration
3	BNU-ESM	China	128*64	College of Global Change and Earth System Science, Beijing Normal University
4	CanESM2	Canada	128*64	Canadian Centre for Climate Modeling and Analysis
5	CCSM4	USA	288*192	National Center for Atmospheric Research, USA
6	CESM1-BGC	USA	288*192	National Science Foundation, Department of Energy, National Center for Atmospheric Research, USA
7	CMCC-CM	Italy	480*240	Centro Euro-Mediterraneo per I Cambiamenti Climatici
8	CMCC-CMS	Italy	192*96	Centro Euro-Mediterraneo per I Cambiamenti Climatici
9	CNRM-CM5	France	256*128	Centre National de Recherches Météorologiques / Centre Européen de Recherche et Formation Avancée en Calcul Scientifique
10	CSIRO-Mk3-6-0	Australia	192*96	Commonwealth Scientific and Industrial Research Organisation in collaboration with the Queensland Climate Change Centre of Excellence
11	FGOALS-g2	China	128*60	LASG, Institute of Atmospheric Physics, Chinese Academy of Sciences and CESS,Tsinghua University
12	GFDL-CM3	USA	144*90	NOAA Geophysical Fluid Dynamics Laboratory
13	GFDL-ESM2G	USA	144*90	Geophysical Fluid Dynamics Laboratory, USA
14	GFDL-ESM2M	USA	144*90	NOAA Geophysical Fluid Dynamics Laboratory, USA
15	GISS-E2-H	USA	144*90	NASA Goddard Institute for Space Studies
16	GISS-E2-R	USA	144*90	NASA Goddard Institute for Space Studies
17	HadGEM2-AO	UK	192*145	National Institute of Meteorological Research/Korea Meteorological Administration
18	HadGEM2-CC	UK	192*145	Met Office Hadley Centre (additional HadGEM2-ES realizations contributed by Instituto Nacional de Pesquisas Espaciais)
19	HadGEM2-ES	UK	192*145	Met. Office Hadley Centre, UK
20	INMCM4	Russia	180*120	Institute for Numerical Mathematics
21	IPSL-CM5A-LR	France	96*96	Institut Pierre-Simon Laplace
22	IPSL-CM5A-MR	France	144*142	Institut Pierre-Simon Laplace
23	IPSL-CM5B-LR	France	96*96	Institut Pierre-Simon Laplace
24	MIROC5	Japan	256*128	Atmosphere and Ocean Research Institute, the University of Tokyo
25	MIROC-ESM	Japan	128*64	Atmosphere and Ocean Research Institute (The University of Tokyo), National Institute for Environmental Studies, and Japan Agency for Marine-Earth Science and Technology
26	MPI-ESM-LR	Germany	192*96	Max Planck Institute for Meteorology (MPI-M)
27	MPI-ESM-MR	Norway	192*96	Max Planck Institute for Meteorology, Germany
28	MRI-CGCM3	Japan	320*160	Meteorological Research Institute
29	NorESM1-M	Norway	144*96	Norwegian Climate Centre, Norway

### Field data collection

The study took place in a Nitrate-vulnerable zone (NVZ) according to the European Nitrate Directive 91/676 [41]. NVZ relates to both the high permeability of the area’s sandy soils which have little potential to retain N and the conventional local agricultural practice in which irrigation and N amendments (mainly manure, slurry and inorganic N fertilizers) are applied at high rates. This combination results in leaching of high levels of NO_3_^-^ to the aquifer [8]. Initial chemical and physical characteristics of soils in the study area were determined from samples collected June 3, 2010, before fertilization or sowing. These values were used as initial inputs for the crop simulation model. Additional soil and crop samples were taken at various growth stages throughout the maize-triticale rotation to measure total soil nitrogen (N), organic carbon (OC), NO_3_^-^ and ammonium (NH_4_^+^) concentrations, as well as total crop N content and biomass production. Soil and crop samples were collected at every sampling date from three replicates for each of the three treatments (N0, NMIN, CONV).

A total of eight soil depths were sampled at 10 cm increments for the first two layers (0–10, 10–20 cm) and at 20 cm increments for the other six layers to a depth of 140 cm (20–40, 40–60, 60–80, 80–100, 100–120, 120–140 cm). For each sampling point, all the plants growing along a one meter length were removed from the field and analyzed. Slurry samples were collected in April 2010 and 2011 with a NISKIN bottle (0.8 m height, 0.07 m dia.), which was specifically designed for sampling liquids at a given depth. Soil and slurry samples were stored in a deep freeze at −20°C until analysis.

Soil texture was determined using the modified pipette procedure for particle-size analysis [42, 43] and organic carbon was determined with the Walkley–Black method [44]. Total N was determined with the Kjeldahl method; NO_3_^-^ was measured by extracting each sample with NaHCO_3_ 0.01 M (weight/vol 1/20) and quantified with the Fox and Piekielek method [45]. Ammonium (NH_4_^+^) was determined by KCl 2M extraction and colorimetric quantification [46]. A pH meter (GLP 21, CRISON, 08328 Alella, Barcelona, Spain), calibrated with pH 4.0 and 7.0 buffer solutions, was used to analyze pH in water samples. Available soil P was determined using the Olsen et al. method [47], and K was determined using a BaCl_2_ and triethanolamine solution [48].

### Crop simulation model

The SALUS model (System Approach to Land Use Sustainability [5,10,30,33,49,50]) used in this study has previously been calibrated and tested on field data collected in the same area of Sardinia at a different location [8]. This study further evaluates SALUS with maize yield and soil NO_3_^-^ levels under a maize-triticale rotation.

SALUS, derived from the CERES models, was designed to simulate, in a continuous mode [33], the long-term impact of management, soil and climate, on crop yield and the environmental impact of cropping systems. SALUS represents an advancement of the CERES models in that it includes several algorithms that improve the simulation of water balance, soil carbon dynamics and crop phenology [33, 51, 52]. SALUS simulates the daily effects of crop rotations, planting dates, plant populations, irrigation, and fertilizer applications on plant growth and soil conditions and has been tested for crop yield (e.g. [5, 24, 53]), soil C dynamics (e.g. [50]), plant N uptake and phenology (e.g. [24, 49]), and NO_3_^-^ leaching (e.g. [8]).

### Crop model simulation scenarios

Simulations were performed with a rotational mode approach, which consists of running the model for the entire duration of the scenario without annual re-initialization of soil parameters. This method makes it possible to fully account for any carry-over effects of water and nutrients that may occur from one year to the next [54]. Crop rotation simulations were performed under the following guidelines:

○ A first set of rotational simulations was carried out for the experimental years to evaluate the model’s ability to simulate maize yield and soil N in each treatment.
○ A second set of rotational simulations was carried out for a long-term assessment of the treatments, using future climate scenarios on the three treatments and on a best management practice (BMP) that was defined in this study. The BMP replicated the use of both slurry and urea as in CONV, but reduced the N rate by 50 kg N ha^-1^ compared to CONV. The BMP consisted of a total N fertilization rate of 223 kg N ha^-1^ year^−1^ (synthetic + organic) with 173 kg N ha^-1^ applied as liquid manure before sowing (DOY 162) and 50 kg N ha^-1^ applied as urea during the growing season (DOY 189). This N rate was determined as optimal based on crop N uptake values observed during the 2010–2011 growing seasons and was designed to comply with the limits imposed by NVZ regulation (170 kg N ha^−1^ year^−1^ from organic amendments).

### Statistical analyses

The ability of the model to predict grain yield was evaluated using the root mean square error (RMSE) as calculated with the following equation:

$$RMSE=\sqrt{\frac{\sum {\left(Obs-Sim\right)}^{2}}{N}}$$ (1)

where *N* is the total number of observations (yield measurements at the end of each season), *Obs* are the observed values, and *Sim* are the simulated values. The relative error (R.E.) between the mean of simulated values and the mean of observed values was calculated to determine how closely the simulation matched the observed mean:

$$R.E.(\%)=\frac{\left|Sim-Obs\right|}{Obs}\times 100$$ (2)

where *Sim* is the simulated value and *Obs* is the mean of observed values. Nitrogen use efficiency (NUE) was calculated with the partial balance approach. Inputs to this method include grain yield, percent moisture, crop N content, and the amount of N applied. NUE was calculated as follows:

$$NUE=\frac{Yield\left(kg.ha^{-1}\right)}{N_{app}}$$ (3)

where *N*_*app*_ is the amount of N fertilizer applied per hectare.

Nitrogen fertilizer efficiency (NFE) was calculated as follows:

$$NFE=\frac{N_{up}}{N_{app}}$$ (4)

where *N*_*up*_ is the crop N uptake.

N fertilizer recovery (*NFrec*) was calculated using the difference method, which is the difference between the N uptake simulated in a given fertilised treatment (*N*_*up*_*)* and in the unfertilised treatments (*N*_*up*,*N0*_), divided by the amount of N applied in the given treatment (*∆N*):

$$NFrec=\frac{N_{up}-N_{up,N0}}{\Delta N}$$ (5)

## Results

### Climate data

**Fig 1:**
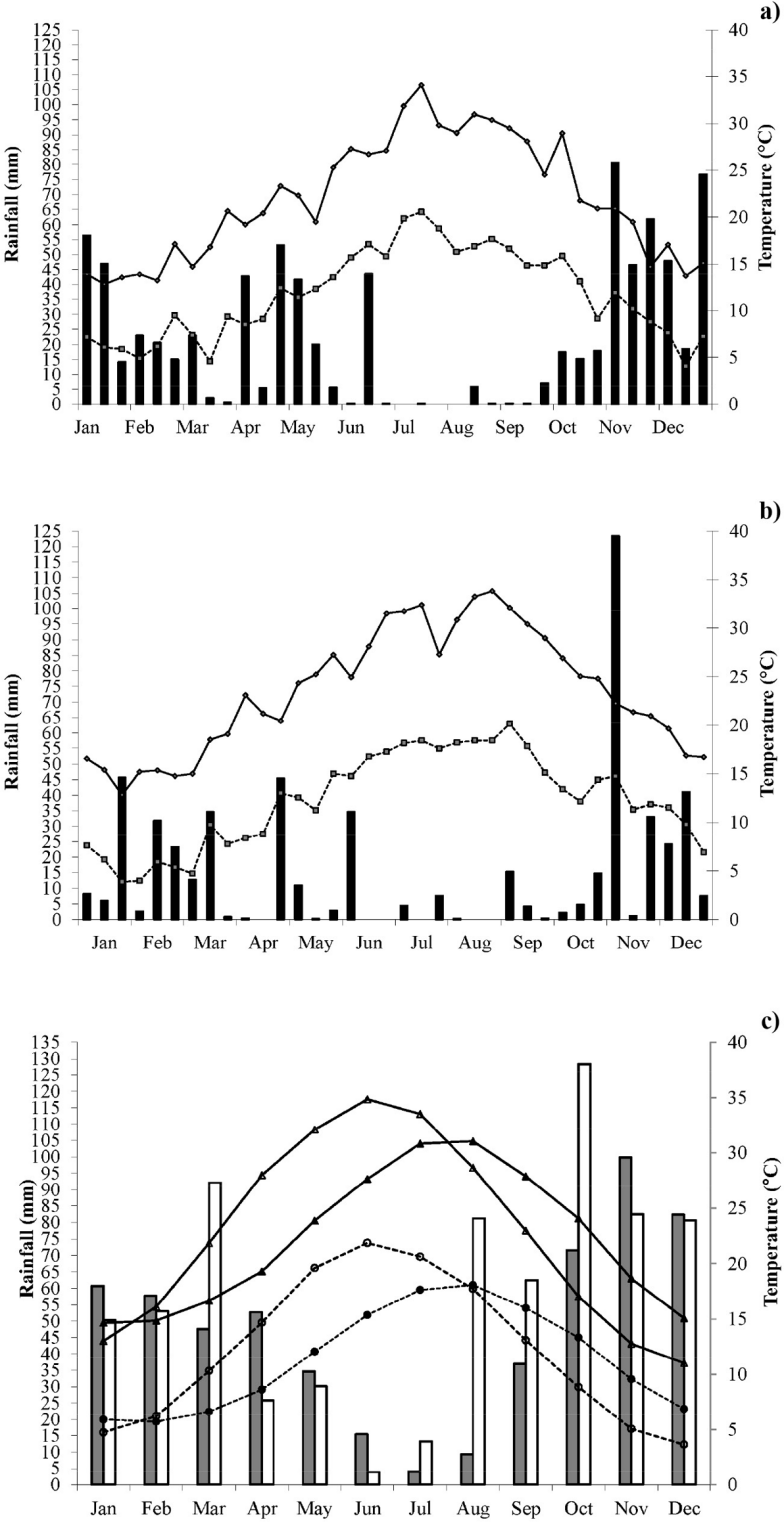
Growing season climate of the study site. Rainfall (bars), maximum (solid line) and minimum (dashed line) temperatures in the years 2010 (a) and 2011 (b). Rainfall (grey bars), maximum (solid line and filled triangle), minimum (dashed line and filled circle) temperatures for fifty-three years: from 1 January 1959 to 31 December 2011 (c). Rainfall (white bars), maximum (solid line and open triangle), minimum (dashed line and open circle) temperatures for eighty-four years of future climate: from 1 January 2012 to 31 December 2095 (c). Rainfall values are sums (a and b) and 84 years average of monthly sums (c); temperature values are means, over 10-day periods (a and b) and 84 years average of monthly means (c).

Climate data for 2010 and 2011 showed that the annual rainfall for these two years was highly variable (Fig 1A and 1B). Annual rainfall in 2010 was 811.2 mm with a maximum of 89 mm in November and a minimum of 0.2 mm in July, while annual rainfall in 2011 was 544 mm, with a maximum of 123 mm in November and a minimum of 0.2 mm in August. Overall, annual rainfall in 2010 was similar to the long-term historic mean, while rainfall in 2011 was below the 1959–2011 historic average (Fig 1C). Temperatures in both years were close to the long-term historic means. Compared to long-term historic means, simulated projections highlighted higher rainfall in March, August, September and October, while projected temperatures were higher from February to August and lower during the remainder of the year (Fig 1C).

### Experimental and modelled data

The results of initial soil chemical and physical analyses are reported in Table 3. The top 40 cm of the soil profile is characterized by very high sand content, (mean value 97.3%), and high concentrations of both OC (20.5 g kg^-1^) and total N (2 g kg^-1^). Average OC at the beginning of the experiment was 8.3 g kg^-1^over the entire soil profile and the average total N was 0.98 g kg^-1^ (Tables 4 and 5). Mean pH was 7.5. The soil had an adequate supply of K_2_O and high levels of P_2_O_5_, especially in the top 60 cm (Table 4). The soil profile NO_3_^-^ and NH_4_^+^ content were, on average, 26.8 and 47.7 mg kg^-1^, respectively (Table 3).

**Table 3:** Soil physical and chemical characteristics for each layer of the soil profile (means and standard errors, n = 3). Soil samples were collected June 3, 2010, before fertilization and sowing.

Depth	Stones (>2mm)	Clay (<0.002 mm)	Silt (0.02–0.002mm)	Sand (2–0.02 mm)	Bulk density	pH	Total N
(m)	(%)	(%)	(%)	(%)	(g cm^-3^)		(g kg^-1^)
0.10	1.8 ± 0.5	1.7 ± 0.3	1.0 ± 0.1	97.3 ± 0.4	1.50 ± 0.012	7.2 ± 0.05	2.1 ± 0.1
0.20	1.5 ± 0.3	1.9 ± 0.1	1.1 ± 0.1	97.0 ± 0.3	1.51 ± 0.010	7.1 ± 0.12	2.0 ± 0.1
0.40	1.6 ± 0.4	2.1 ± 0.3	1.4 ± 0.1	96.5 ± 0.2	1.51 ± 0.007	7.1 ± 0.13	1.9 ± 0.1
0.60	1.7 ± 0.5	1.5 ± 0.1	1.2 ± 0.2	97.3 ± 0.3	1.61 ± 0.007	7.3 ± 0.06	1.1 ± 0.0
0.80	2.5 ± 0.6	0.7 ± 0.3	0.9 ± 0.1	98.4 ± 0.3	1.67 ± 0.004	7.6 ± 0.11	0.5 ± 0.0
1.00	1.1 ± 0.4	1.7 ± 0.8	0.9 ± 0.1	97.5 ± 0.8	1.67 ± 0.011	7.7 ± 0.16	0.5 ± 0.1
1.20	1.9 ± 0.7	2.5 ± 0.8	0.6 ± 0.1	97.0 ± 0.8	1.66 ± 0.010	7.7 ± 0.12	0.4 ± 0.0
1.40	1.8 ± 0.6	2.2 ± 0.3	0.6 ± 0.1	97.3 ± 0.3	1.67 ± 0.004	7.8 ± 0.13	0.4 ± 0.0
Depth	NO_3_^-^	NH_4_^+^	Organic carbon	C/N	P_2_O_5_	K_2_O	
(m)	(mg kg^-1^)	(mg kg^-1^)	(g kg^-1^)		(mg kg^-1^)	(mg kg^-1^)	
0.10	22.9 ± 5.6	49.1 ± 7.1	21.8 ± 1.0	10.2 ± 0.1	189.6 ± 14.7	97.5 ± 21.4	
0.20	32.0 ± 9.7	60.3 ± 13.6	20.1 ± 1.0	10.0 ± 0.1	243.5 ± 38.6	140.3 ± 53.4	
0.40	29.1 ± 5.9	72.4 ± 6.1	20.0 ± 1.0	10.5 ± 0.2	259.4 ± 26.7	106.0 ± 17.0	
0.60	31.2 ± 8.5	59.8 ± 9.9	9.0 ± 1.0	7.9 ± 0.5	196.6 ± 36.5	128.8 ± 27.6	
0.80	21.1 ± 7.1	43.6 ± 11.5	2.7 ± 0.0	5.7 ± 1.2	73.2 ± 16.6	87.3 ± 13.2	
1.00	25.9 ± 6.8	36.7 ± 12.0	2.3 ± 1.0	5.3 ± 1.3	32.0 ± 6.0	129.4 ± 38.2	
1.20	26.2 ± 4.2	32.6 ± 9.1	2.1 ± 0.0	5.2 ± 1.6	23.6 ± 5.2	176.4 ± 43.2	
1.40	26.9 ± 1.7	34.3 ± 8.9	1.3 ± 0.0	3.1 ± 0.3	20.0 ± 3.4	46.5 ± 23.2	

**Table 4:** Means and standard errors of organic carbon content in soil profile layers related to three sampling dates during the maize-triticale-maize rotation for the N MIN, CONV and N0 treatments (n = 3). Sample dates correspond to the harvest of maize (09/14/2010), triticale (05/10/2011) and maize (09/08/2011). Dates are reported as mm/dd/yy.

Sampling dates (mm/dd/yy)	Treatment	Organic carbon (g Kg^-1^)							
		0–10 cm	10–20 cm	20–40 cm	40–60 cm	60–80 cm	80–100 cm	100–120 cm	120–140 cm
09/14/2010	N MIN	32.1 ± 1.2	23.3 ± 0.6	22.9 ± 0.9	16.2 ± 3.1	4.4 ± 0.9	2.8 ± 0.4	2.7 ± 0.5	2.0 ± 0.0
09/14/2010	CONV	26.7 ± 2.2	23.0 ± 0.2	21.0 ± 0.7	15.8 ± 3.0	3.7 ± 0.6	3.4 ± 0.4	3.4 ± 0.4	2.6 ± 0.4
09/14/2010	N 0	28.2 ± 0.8	20.2 ± 1.7	23.4 ± 2.8	18.3 ± 5.3	4.6 ± 0.8	3.0 ± 0.9	2.7 ± 0.6	2.3 ± 0.8
05/10/2011	N MIN	21.8 ± 1.2	19.8 ± 1.0	20.2 ± 0.6	12.3 ± 1.1	2.3 ± 0.4	1.7 ± 0.5	1.5 ± 0.1	1.1 ± 0.4
05/10/2011	CONV	21.5 ± 0.3	19.3 ± 1.3	16.6 ± 1.2	14.1 ± 3.1	3.4 ± 0.5	1.8 ± 0.1	1.6 ± 0.2	0.9 ± 0.3
05/10/2011	N 0	16.8 ± 0.6	18.6 ± 0.7	17.7 ± 0.5	12.0 ± 2.1	3.0 ± 0.9	1.8 ± 0.2	2.0 ± 0.4	0.9 ± 0.4
09/08/2011	N MIN	31.2 ± 0.5	30.2 ± 0.6	33.0 ± 0.4	25.9 ± 1.4	5.2 ± 1.4	2.6 ± 0.1	1.9 ± 0.2	2.2 ± 0.1
09/08/2011	CONV	32.7 ± 0.7	29.2 ± 2.6	31.9 ± 2.0	18.1 ± 2.5	3.4 ± 0.1	2.3 ± 0.2	2.3 ± 0.2	2.6 ± 0.1
09/08/2011	N 0	27.2 ± 1.1	25.7 ± 2.0	30.4 ± 3.5	22.9 ± 0.8	6.2 ± 0.6	3.0 ± 0.1	2.1 ± 0.3	1.8 ± 0.2

**Table 5:** Means and standard errors of total N content in soil profile layers related to three sampling dates during the maize-triticale-maize rotation for the N MIN, CONV and N0 treatments (n = 3). Sample dates correspond to the harvest of maize (09/14/2010), triticale (05/10/2011) and maize (09/08/2011). Dates are reported as mm/dd/yy.

Sampling dates (mm/dd/yy)	Treatment	Total Nitrogen (g Kg^-1^)							
		0–10 cm	10–20 cm	20–40 cm	40–60 cm	60–80 cm	80–100 cm	100–120 cm	120–140 cm
09/14/2010	N MIN	2.8 ± 0.20	2.2 ± 0.10	2.2 ± 0.10	1.5 ± 0.30	0.4 ± 0.09	0.3 ± 0.06	0.3 ± 0.06	0.2 ± 0.03
09/14/2010	CONV	2.5 ± 0.30	2.2 ± 0.10	1.6 ± 0.20	1.3 ± 0.30	0.3 ± 0.03	0.3 ± 0.03	0.3 ± 0.07	0.2 ± 0.03
09/14/2010	N 0	2.6 ± 0.20	1.9 ± 0.20	2.2 ± 0.30	1.4 ± 0.30	0.4 ± 0.03	0.4 ± 0.07	0.3 ± 0.00	0.2 ± 0.03
05/10/2011	N MIN	1.9 ± 0.10	1.9 ± 0.10	1.8 ± 0.09	1.0 ± 0.09	0.3 ± 0.03	0.2 ± 0.03	0.2 ± 0.03	0.1 ± 0.03
05/10/2011	CONV	1.8 ± 0.06	1.8 ± 0.18	1.5 ± 0.17	1.2 ± 0.29	0.3 ± 0.06	0.1 ± 0.03	0.1 ± 0.06	0.1 ± 0.07
05/10/2011	N 0	1.6 ± 0.12	1.6 ± 0.06	1.6 ± 0.10	1.1 ± 0.18	0.4 ± 0.03	0.2 ± 0.06	0.2 ± 0.00	0.1 ± 0.00
09/08/2011	N MIN	2.7 ± 0.12	2.7 ± 0.06	2.9 ± 0.12	2.1 ± 0.12	0.5 ± 0.06	0.3 ± 0.00	0.2 ± 0.03	0.2 ± 0.00
09/08/2011	CONV	2.7 ± 0.18	2.4 ± 0.20	2.7 ± 0.12	1.5 ± 0.18	0.3 ± 0.00	0.2 ± 0.03	0.3 ± 0.00	0.2 ± 0.03
09/08/2011	N 0	2.3 ± 0.13	2.3 ± 0.09	2.6 ± 0.15	1.9 ± 0.06	0.5 ± 0.06	0.3 ± 0.03	0.2 ± 0.03	0.2 ± 0.00

OC and total N content were measured again when maize and triticale were harvested. These results are summarized in Tables 4 and 5. Average OC levels for both maize harvest dates for NMIN, CONV and N0 were 11.2, 10.7, and 11.2 g kg^-1^ in 2010 and 14.5, 13.1, and 13.3 g kg^-1^ in 2011 (Table 4). At the triticale harvest date (05/10/2011), average soil profile OC levels were 8.6 g kg^-1^ (NMIN), 8.4 g kg^-1^ (CONV), and 7.9 g kg^-1^ (N0) (Table 4). The three treatments under triticale showed values closer to the OC observed at the beginning of the experiment (8.3 g kg^-1^), while after maize was harvested, higher values were observed (Table 4). Total N, as the average for the soil profile for maize at harvest time, were 1.1, 0.9, and 1.0 g kg^-1^ for NMIN, CONV, and N0 in 2010 and 1.3, 1.1, and 1.2 g kg^-1^ for NMIN, CONV, and N0 in 2011 (Table 5). At the harvest date of triticale, the values for total N, as an average for the soil profile, were 0.80, 0.73, and 0.74 g kg^-1^ for NMIN, CONV, and N0. Concentrations of OC and total N measured for triticale were lower than the values observed for maize for all three treatments. This was observed both in the soil profile average, and for each of the soil layers. Overall, values for soil OC and N for triticale were about 32% lower than those for maize.

NO_3_^-^ and NH_4_^+^ concentrations in the soil profile were measured at various dates during the experiment and the results are shown as the means for the soil profile (0–140 cm) in Table 6. NO_3_^-^ concentrations ranged between 13.6 and 43.1 mg kg^-1^ for NMIN, 13.8 and 47.6 mg kg^-1^ for CONV, and 9.3 and 30.6 mg kg^-1^ for N0 (Table 6). Ammonium concentrations ranged between 11.9 and 51.3 mg kg^-1^ for NMIN, 4.3 and 81.6 mg kg^-1^ for CONV, and 8.8 and 33.0 mg kg^-1^ for N0 (Table 6). Within each treatment, the concentration of both NO_3_^-^ and NH_4_^+^ varied greatly and values obtained during the triticale growing season were lower than those that were measured at the beginning of the experiment (Tables 3 and 6).

**Table 6:** Measured means and standard errors of nitrate and ammonium soil profile content during the maize-triticale-maize rotation for the N MIN, CONV and N0 treatments (n = 3). Values of the eight soil depths were averaged for each sample and the standard errors calculated for the three replicates. Dates are reported as mm/dd/yy.

Sampling dates	Treatment	Depths	NO_3_^-^	NH_4_^+^
(mm/dd/yy)		(m)	(mg kg^-1^)	(mg kg^-1^)
07/07/2010	N MIN	0–1.40	36.4 ± 7.1	51.3 ± 23.9
07/07/2010	CONV	0–1.40	31.4 ± 7.3	81.6 ± 16.7
07/07/2010	N0	0–1.40	23.1 ± 2.8	33.0 ± 2.4
07/30/2010	N MIN	0–1.40	43.1 ± 17.6	21.9 ± 3.5
07/30/2010	CONV	0–1.40	27.3 ± 8.0	22.4 ± 3.1
07/30/2010	N0	0–1.40	15.9 ± 2.3	32.2 ± 3.7
08/18/2010	N MIN	0–1.40	24.4 ± 4.7	26.1 ± 3.5
08/18/2010	CONV	0–1.40	32.1 ± 3.2	18.9 ± 3.8
08/18/2010	N0	0–1.40	21.2 ± 2.5	14.1 ± 2.5
09/14/2010	N MIN	0–1.40	21.3 ± 2.6	11.9 ± 2.5
09/14/2010	CONV	0–1.40	27.2 ± 3.3	9.4 ± 2.0
09/14/2010	N0	0–1.40	30.6 ± 3.2	19.6 ± 2.4
02/09/2011	N MIN	0–1.40	26.4 ± 3.5	36.8 ± 4.6
02/09/2011	CONV	0–1.40	24.3 ± 2.3	18.0 ± 3.5
02/09/2011	N0	0–1.40	21.6 ± 2.2	29.7 ± 3.5
04/06/2011	N MIN	0–1.40	14.4 ± 1.0	12.9 ± 1.2
04/06/2011	CONV	0–1.40	14.4 ± 4.3	20.7 ± 6.2
04/06/2011	N0	0–1.40	10.6 ± 0.9	16.1 ± 2.9
05/10/2011	N MIN	0–1.40	16.1 ± 0.5	22.4 ± 3.6
05/10/2011	CONV	0–1.40	13.8 ± 0.5	14.1 ± 2.0
05/10/2011	N0	0–1.40	9.3 ± 0.9	13.5 ± 2.7
06/22/2011	N MIN	0–1.40	35.0 ± 9.7	24.1 ± 9.1
06/22/2011	CONV	0–1.40	35.6 ± 8.1	16.8 ± 11.6
06/22/2011	N0	0–1.40	24.0 ± 3.8	14.3 ± 2.7
07/06/2011	N MIN	0–1.40	37.0 ± 13.4	26.3 ± 4.9
07/06/2011	CONV	0–1.40	47.6 ± 18.3	33.0 ± 7.2
07/06/2011	N0	0–1.40	22.8 ± 2.1	8.8 ± 1.8
09/08/2011	N MIN	0–1.40	13.6 ± 1.7	13.8 ± 1.0
09/08/2011	CONV	0–1.40	18.9 ± 2.0	4.3 ± 0.5
09/08/2011	N0	0–1.40	14.7 ± 1.4	8.9 ± 1.3

Crop biomass, grain and stover N contents, and crop N uptake were measured in each treatment at harvest (Table 7). No results are provided for the N content in the triticale grain since triticale was harvested for silage. Overall, the N0 treatment showed the lowest values for all the variables evaluated, whereas similar values for all variables were observed in the NMIN and CONV treatments (Table 7). Crop N uptake for triticale was higher for NMIN than for the other two treatments (Table 7).

**Table 7:** Means and standard errors of biomass, N content and N uptake at the harvest dates of maize and triticale crops (n = 3). Sampling dates correspond to the harvest of maize (09/14/2010), triticale (05/10/2011) and maize (09/08/2011). Dates are reported as mm/dd/yy.

Crop	Sampling dates	Treatment	Biomass	N content stover	N content grain	N uptake
	(mm/dd/yy)		(t ha^-1^)	(g kg^-1^)	(g kg^-1^)	(kg ha^-1^)
	09/14/2010	N MIN	23.68 ± 1.64	9.5 ± 0.6	14.5 ± 0.4	267.9 ± 26.9
Maize	09/14/2010	CONV	22.45 ± 1.86	12.6 ± 0.4	14.6 ± 0.4	280.6 ± 16.8
	09/14/2010	N 0	20.22 ± 1.65	7.0 ± 0.6	10.8 ± 0.4	143.0 ± 22.0
	05/10/2011	N MIN	7.50 ± 1.08	14.3 ± 0.6	-	131.4 ± 13.3
Triticale	05/10/2011	CONV	7.91 ± 0.58	11.4 ± 0.5	-	90.5 ± 8.4
	05/10/2011	N 0	6.80 ± 1.96	7.7 ± 0.4	-	53.8 ± 17.6
	09/08/2011	N MIN	25.50 ± 0.43	6.6 ± 0.6	11.3 ± 0.5	232.3 ± 12.3
Maize	09/08/2011	CONV	25.63 ± 0.61	6.5 ± 0.6	11.2 ± 0.4	229.8 ± 13.3
	09/08/2011	N 0	12.72 ± 0.78	2.6 ± 0.2	8.4 ± 0.4	62.0 ± 3.2

The model evaluation of maize yield for both growing seasons (2010 and 2011) and for the three N treatments is shown in Table 8. The observed maize yields in 2010 were 23.68, 22.45 and 20.22 t ha^-1^ for NMIN, CONV, and N0, respectively (Table 8). In 2011, observed yields were 25.50t ha^-1^ for NMIN, 25.6t ha^-1^ for CONV and 12.7t ha^-1^ for N0. Overall, SALUS effectively reproduced the observed yield for each treatment. RMSE values for the harvested grain during the two cropping seasons varied from 0.73 t ha^-1^ to 4.10 t ha^-1^ in NMIN, from 0.45 t ha^-1^ to 3.53 t ha^-1^ in CONV, and from 0.10 t ha^-1^ to 0.12 t ha^-1^ in N0 (Table 8). The R.E. of observed and simulated grain yield ranged from 3.08% to 16.08% in NMIN, from 2% to 13.77% in CONV, and from 0.49% to 0.94% in N0 (Table 8).

**Table 8:** Observed (means and standard errors, n = 3) and simulated silage maize yields for the NMIN, CONV and N0 treatments. Yields are only reported for maize as triticale was harvested for silage. Sampling dates correspond to the harvest of maize.

Date	Treatment	Yield (t ha^-1^)		RMSE	R.E.
(mm/dd/yy)		Observed	Simulated	(t ha ^-1^)	(%)
09/14/2010	N MIN	23.68 ± 1.64	22.95	0.73	3.08
09/14/2010	CONV	22.45 ± 1.86	22.00	0.45	2.00
09/14/2010	N 0	20.22 ± 1.65	20.12	0.10	0.49
09/08/2011	N MIN	25.50 ± 0.43	21.40	4.10	16.08
09/08/2011	CONV	25.63 ± 0.61	22.10	3.53	13.77
09/08/2011	N 0	12.72 ± 0.78	12.60	0.12	0.94

**Fig 2:**
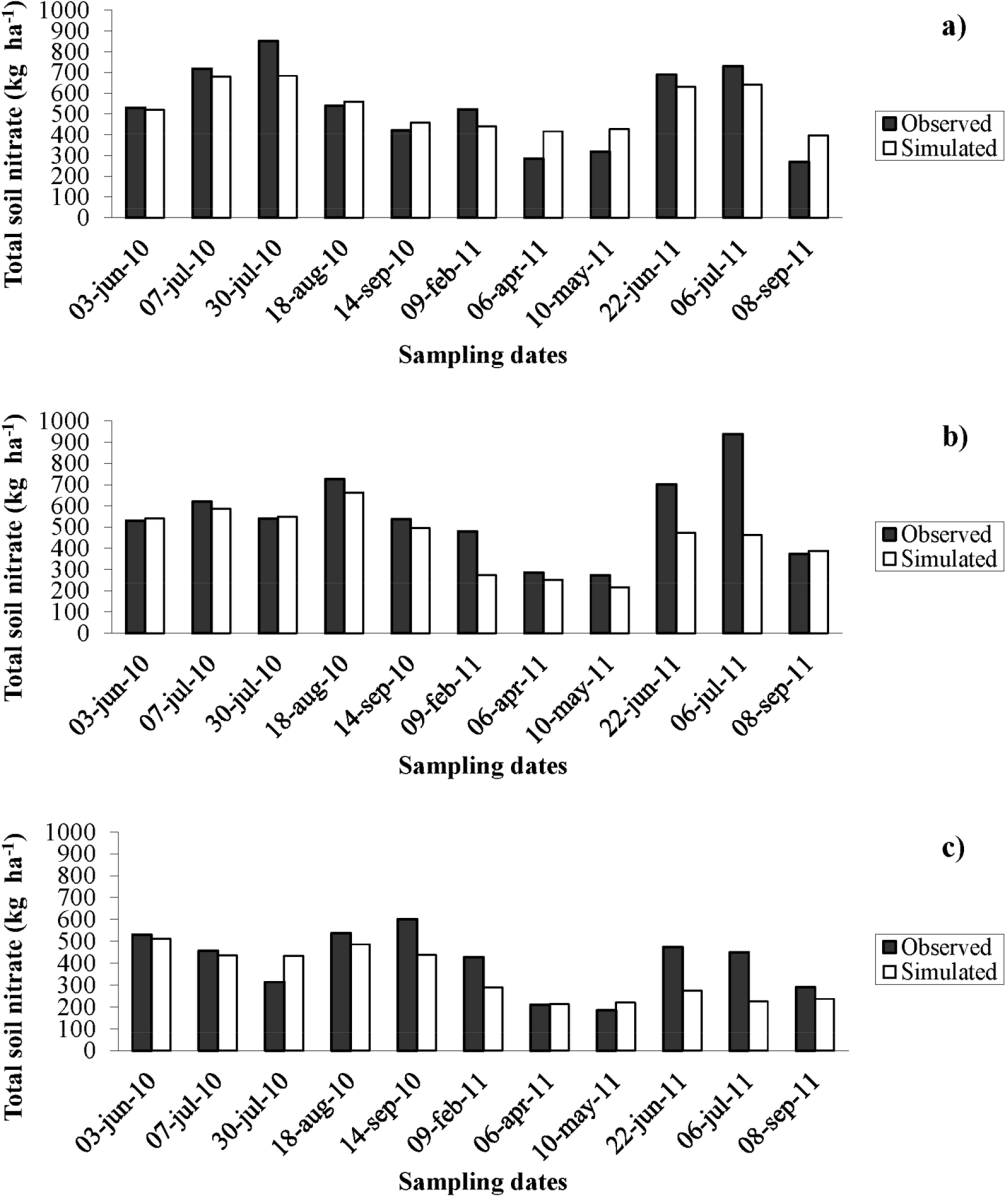
Observed and simulated soil profile nitrate content. Observed (filled bars) and simulated (open bars) soil profile nitrate (NO3-) content during the maize-triticale-maize rotation for the NMIN (a), CONV (b) and N0 (c) treatments.

**Fig 3:**
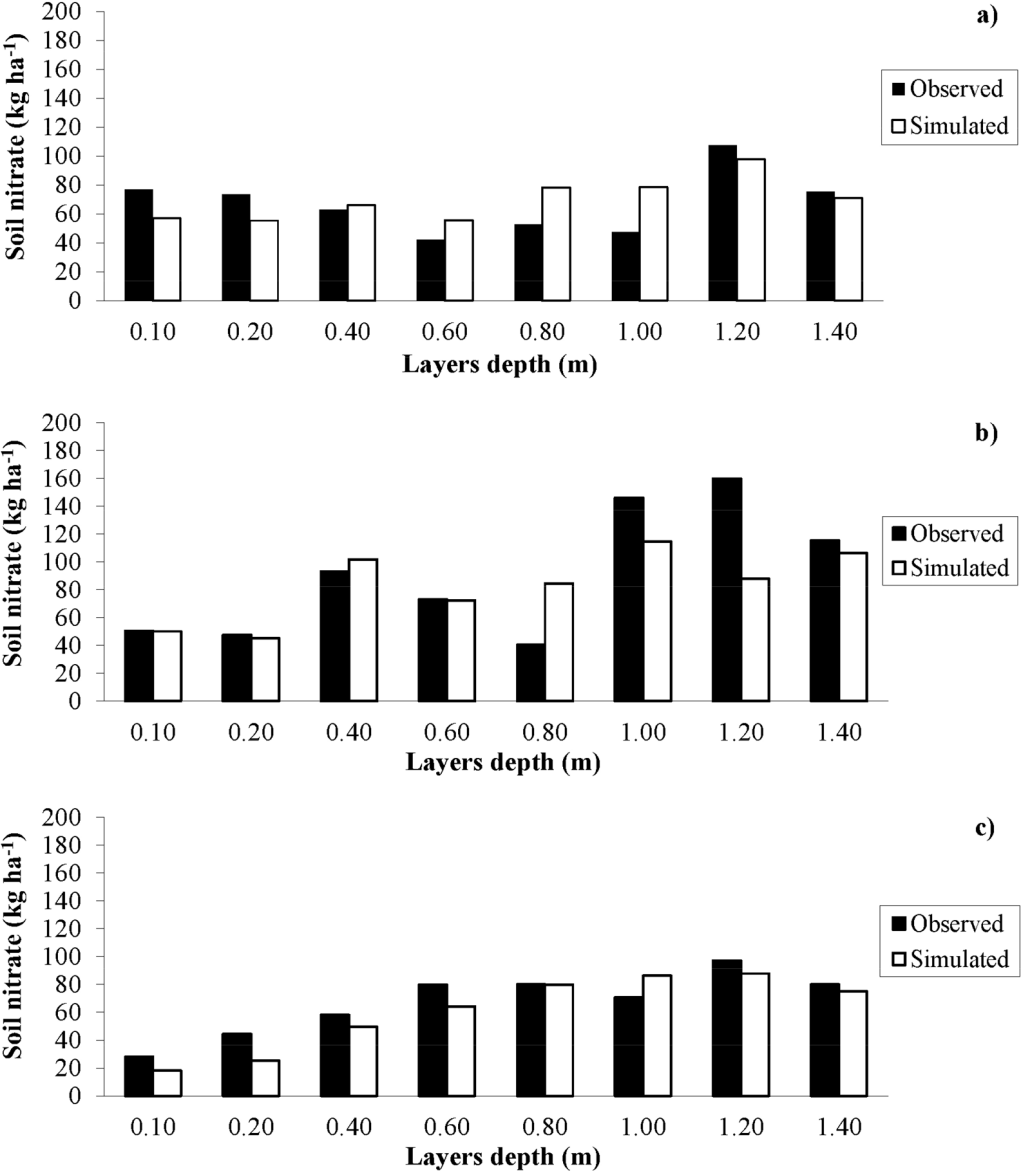
Observed and simulated soil profile nitrate content. Observed (filled bars) and simulated (open bars) nitrate (NO3-) content in soil profile layers for the NMIN (a), CONV (b) and N0 (c) treatments on August 18, 2010.

The SALUS model also effectively reproduced the magnitude and the temporal patterns of the soil profile NO_3_^-^ over the crop rotation (Fig 2). The total soil profile NO_3_^-^ content under CONV treatment was well-simulated, except for four dates when it was underestimated by an amount that ranged from 62.7 to 476.9 kg NO_3_^-^ ha^-1^ (see Figs 2B and 3B). The total soil profile NO_3_^-^ content for the N0 treatment was overestimated by 120 kg NO_3_^-^ ha^-1^ on only one date and was underestimated for five dates by amounts that ranged from 51.8 to 225.4 NO_3_^-^ ha^-1^ (see Figs 2C and 3C).

The underestimation of total NO_3_^-^ concentrations on some dates was mainly due to the simulation of low NO_3_^-^ levels in the lower layers of the soil profile. This can be observed in Fig 3, where NO_3_^-^ levels are reported for each soil layer on August 18 2010, when NO_3_^-^ levels were underestimated in all three treatments. On this date, simulation of the NMIN treatment highlighted some slight overestimation at 0.6 m (by 13.2 kg NO_3_^-^ ha^-1^), at 0.8m (by 25.8 kg NO_3_^-^ ha^-1^), and at 1 m (by 31 kg NO_3_^-^ ha^-1^), and moderate underestimation at 0.1, 0.2 and 1.2 m by 19.9, 18 and 9.7 kg NO_3_^-^ ha^-1^ (Fig 3A). For the CONV treatment, soil NO_3_^-^ levels were overestimated at 0.8 m by 44 kg NO_3_^-^ ha^-1^ and underestimated at 1 m and 1.2 m by 31.2 and 71.9 kg NO_3_^-^ ha^-1^, respectively (Fig 3B). The soil NO_3_^-^ for N0 were overestimated at 1 m by 15.7 kg NO_3_^-^ ha^-1^, and slightly underestimated at 0.1, 0.2, 0.4, 0.6 and 1.2 m by 10, 19, 8.7, 15.7 and 9.2 kg NO_3_^-^ ha^-1^, respectively (Fig 3C).

### Rotational scenarios simulation results

The comparison between BL and the two RCPs highlighted minor differences between the three scenarios, while the coefficient of variation (the ratio between the daily standard deviation over the 29 GCMs and the daily mean value) varied depending on the specific parameter measured. On average, the difference in temperature equaled +1.4°C between the historic baseline scenario (BL—1959–2013) and the RCP6.0 emission scenario and +2.0°C between BL and RCP8.5. The coefficient of variation (CV) between BL and the two emission scenarios equaled 4% for RCP6.0 and 6% for RCP8.5. The CV tended to be constant and independent from the magnitude of the temperature. The average difference between BL and the two RCPs in terms of solar radiation was approximately null (0.04 MJ m^-2^ d^-1^ and 0.06 MJ m^-2^ d^-1^, respectively), and was characterized by a very small CV of 0.025 MJ.m^-2^ d^-1^ for RCP6.0 and 0.04 MJ.m^-2^ d^-1^for RCP8.5. Even though the average difference in daily precipitation between BL and the two RCP was approximately null (-0.036 and -0.054 mm d^-1^, respectively), its CV over the GCMs was larger (25% for RCP6.0 and 41% for RCP8.5). The higher rain events were characterized by the highest variability over the 29 GCMs as the standard error increased with the absolute precipitation amount.

**Fig 4:**
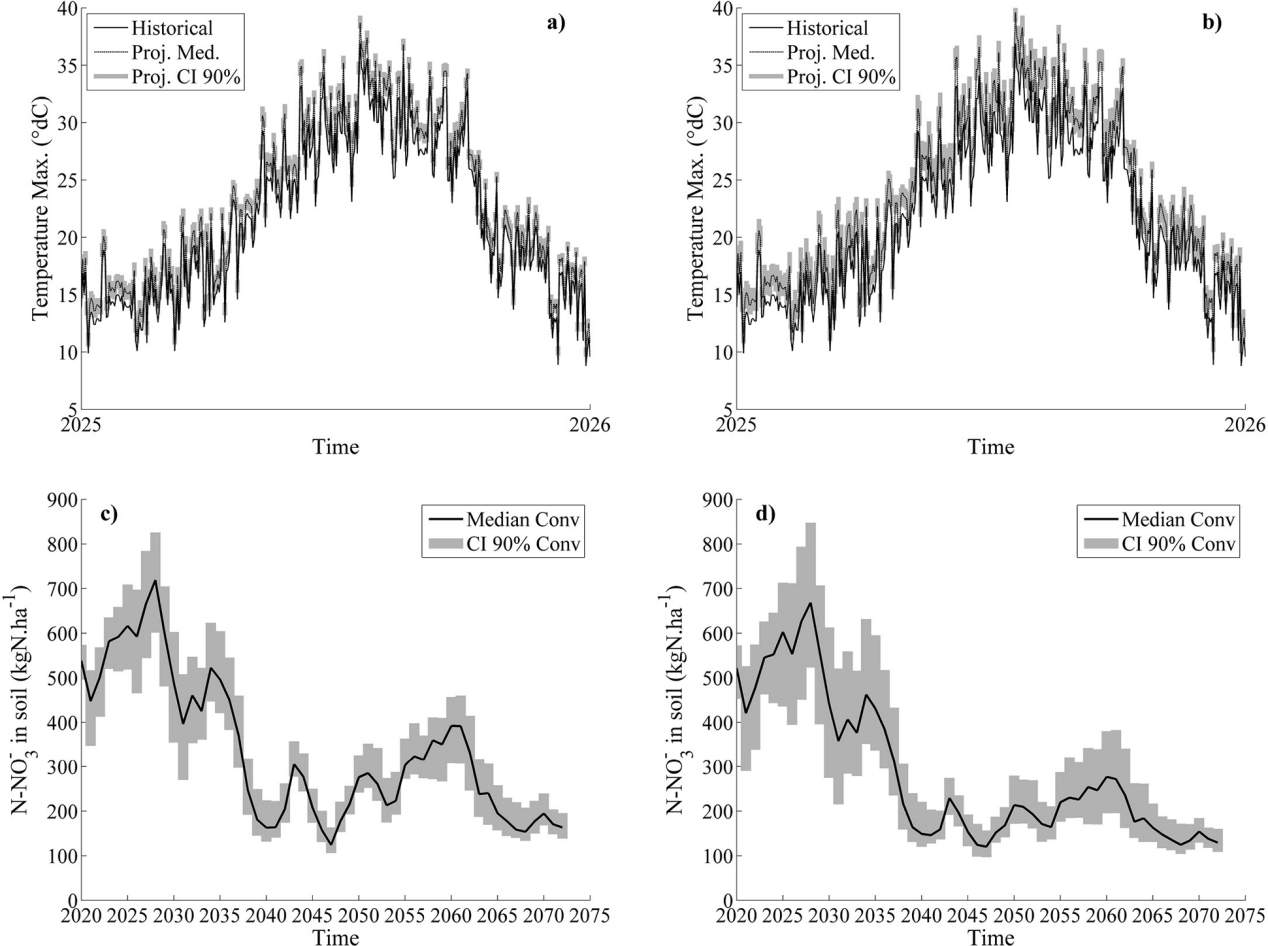
Daily observed and projected maximal temperature under two different emission scenarios and projected impacts on soil nitrogen content. Daily maximal temperature observed (black line) and projected (dashed black line) under emission scenario RCP6.0 (a) and RCP 8.5 (b) for the year 2025. Simulated annual median soil N-NO3- content (black line) for CONV treatment and under emission scenario RCP 6.0 (c) and RCP 8.5 (d). The confidence interval (CI) drawn out of the 29 GCMs is represented by the shaded grey area.

Limited variations in daily maximal temperatures were observed between emission scenarios when downscaling the climatic conditions with the 29 GCMs. This is shown in Fig 4A and 4B, where intra-annual variability of maximal temperatures is shown for one sample year (2025) to improve readability. Simulation of long-term soil NO_3_^-^ dynamics resulting from the 29 GCMs showed lower average NO_3_^-^ contents under RCP6.0, while the RCP 8.5 emission scenario was characterized by higher inter-annual variability (Fig 4C and 4D).

Each of the simulations obtained with a different GCM was compared to the simulation obtained with the BL scenario. For each emission scenario the differences were then averaged and the standard errors computed using a yearly average. The CVs were then computed to express the variability due to the choice of GCMs, as a percentage of the average difference between BL and the considered emission scenario.

The average simulated silage yield difference equaled 1440 kg ha^-1^ between BL and RCP6.0 and 3460 kg ha^-1^ between BL and RCP8.5. These differences were characterized by an average standard deviation due to the GCM of 48.9 kg.ha^-1^ (3.4%) and 155 kg.ha^-1^ (4.5%) considering RCP6.0 and RCP8.5, respectively. Differences in projected yields between the BL and the two emission scenarios remained constant across treatments. The slight increase in biomass accumulation under climate change occurred because the crop is harvested as silage, and not for grains, in fact the harvest index is rather lower than the baseline. Annual average N uptake was slightly higher under RCP6.0 (+4.32 to +24.26 kg N ha^-1^) and RCP8.5 (+6.13 to +34.85 kg N ha^-1^) compared to BL scenario (Table 9). BMP treatment showed the lowest increase (+6.13 kg N ha^-1^ under RCP8.5 vs. BL), followed by N0 (+14.95 kg N ha^-1^ under RCP8.5 vs. BL). NMIN and CONV had the highest increase (+32.20 and + 34.85 kg N ha^-1^ under RCP8.5 vs. BL). NMIN exhibited the lowest variability due to GCMs in terms of N uptake difference (on average +- 5.2% under RCP8.5 vs BL) and N0 treatment had the highest variability due to the GCMs (+9.1% under RCP8.5).

**Table 9:** Difference (Δ) in terms of model simulations between historic BL and downscaled climatic data under both RCP6.0 and RCP8.5 emission scenarios. Total biomass, N uptake, and N-NO_3_^-^ leaching are compared for each treatment in the long term rotation (2020–2073). Results report means and coefficients of variation. Positive value indicates that simulation obtained under RCM is superior to simulation obtained under BL scenario.

Treatments	Total N supply	Δ Biomass	Δ N uptake	Δ N-NO_3_^-^ leached
	(kg N ha^-1^)	(kg d.m. ha^-1^)	(kg N ha^-1^)	(kg N ha^-1^)
BL—RCP 6.0NMIN	230	1427 (± 3.40%)	15.41 (± 3.75%)	5.83 (± 25.97%)
CONV	273	1453 (± 3.29%)	24.26 (± 5.80%)	11.57 (± 49.93%)
N0	-	1510 (± 3.38%)	12.52 (± 6.10%)	4.47 (± 32.52%)
BMP	223	1381 (± 3.31%)	4.32 (± 5.58%)	3.81 (± 31.94%)
BL—RCP 8.5				
NMIN	230	3669 (± 4.37%)	34.85 (± 5.23%)	-7.75 (± 40.73%)
CONV	273	3615 (± 4.37%)	32.20 (± 7.65%)	11.57 (± 67.91%)
N0	-	3127 (± 4.75%)	14.95 (± 9.08%)	5.04 (± 49.57%)
BMP	223	3453 (± 4.50%)	6.13 (± 6.82%)	4.20 (± 43.30%)

Differences in NO_3_^-^ annually leached were globally very low, when BL is compared to RCP6.0 and RCP8.5 (Table 9). Results were quite similar under both emissions scenarios. BMP was characterized by the lowest difference in annually leached NO_3_^-^ (+4.0 kg N ha^-1^ on average for RCP6.0 and RCP8.5) and CONV by the highest (+11.5 kg N ha^-1^ on average for RCP6.0 and RCP8.5). NMIN demonstrated a leaching difference of +5.8 kg N ha^-1^ under RCP6.0 but a negative difference of -7.7 kg N ha^-1^ under RCP8.5. While the differences from BL were quite similar for both emission scenarios, the variability of these differences were higher for all treatments under RCP8.5, *i*.*e*. 25–50% under RCP6.0 and 40–67% under RCP8.5 (Fig 4C and 4D). This is thought to be associated to the high variability of downscaled rainy events.

Table 10 shows the simulated yearly averages of maize biomass, N uptake, N use efficiency (NUE), N fertilizer efficiency (NFE) and the % fertilizer recovery for the four management practices (the original three from the field study plus the BMP treatment) using future climate projections under both RCP6.0 and 8.5 emissions scenarios. The medians of model simulations were computed over the 29 GCMs, at daily and seasonal time steps. Table 10 also reports the mean and standard errors over the 53 years of simulations. Under the RCP8.5 emission scenario, simulated maize biomass was 26.3 t ha^-1^ for NMIN, 26.1 t ha^-1^ for CONV, and 25.9 t ha^-1^ for BMP, while simulated biomass for N0 was 23.8 t ha^-1^ (Table 10). Simulated crop N uptake was 372.6, 267.3, 151.1 and 231.8 kg N ha^-1^ for NMIN, CONV, N0, and BMP, respectively (Table 8). Nitrogen use efficiency (NUE) was 114.4 kg kgN^-1^ for NMIN, 95.7 kg kgN^-1^ for CONV, and 116.2 kg kgN^-1^ for BMP, while the fertilizer recovery rates were 95.8% for NMIN, 42.6% for CONV, and 35.8% for BMP (Table 10).

**Table 10:** Simulated yearly averages of total N supply, biomass (expressed as dry matter), N uptake, N use efficiency (NUE), N fertilizer efficiency (NFE) and fertilizer recovery related to the maize crop for the NMIN, CONV, N0 and BMP treatments in the long-term rotation (2020–2073). Results report means and standard errors.

Treatments	Total N supply	Biomass	N uptake	NUE	NFE	Fertilizer recovery
	(kg N ha^-1^)	(t d.m. ha^-1^)	(kg N ha^-1^)	(kg.kg N^-1^)	(%)	(%)
RCP 6.0NMIN	230	24.1 ± 2.3	352.7 ± 32.7	104.6 ± 10.2	153.4 ± 14.2	88.7 ± 24.5
CONV	273	23.9 ± 2.2	259.4 ± 18.2	87.8 ± 8.2	95.0 ± 6.7	40.6 ± 20.1
N0	-	22.2 ± 2.2	148.7 ± 47.5	-	-	-
BMP	223	23.8 ± 2.2	230.0 ± 12.2	106.9 ± 9.8	103.1 ± 5.5	36.5 ± 20.4
RCP 8.5						
NMIN	230	26.3 ± 2.4	371.6 ± 30.2	114.2 ± 10.5	161.6 ± 13.1	95.7 ± 22.7
CONV	273	26.1 ± 2.3	267.3 ± 18.7	95.7 ± 8.4	97.9 ± 6.8	42.6 ± 21.3
N0	-	23.8 ± 2.4	151.1 ± 50.2	-	-	-
BMP	223	25.9 ± 2.2	231.8 ± 11.5	116.2 ± 10.0	103.9 ± 5.2	35.8 ± 21.7

**Fig 5:**
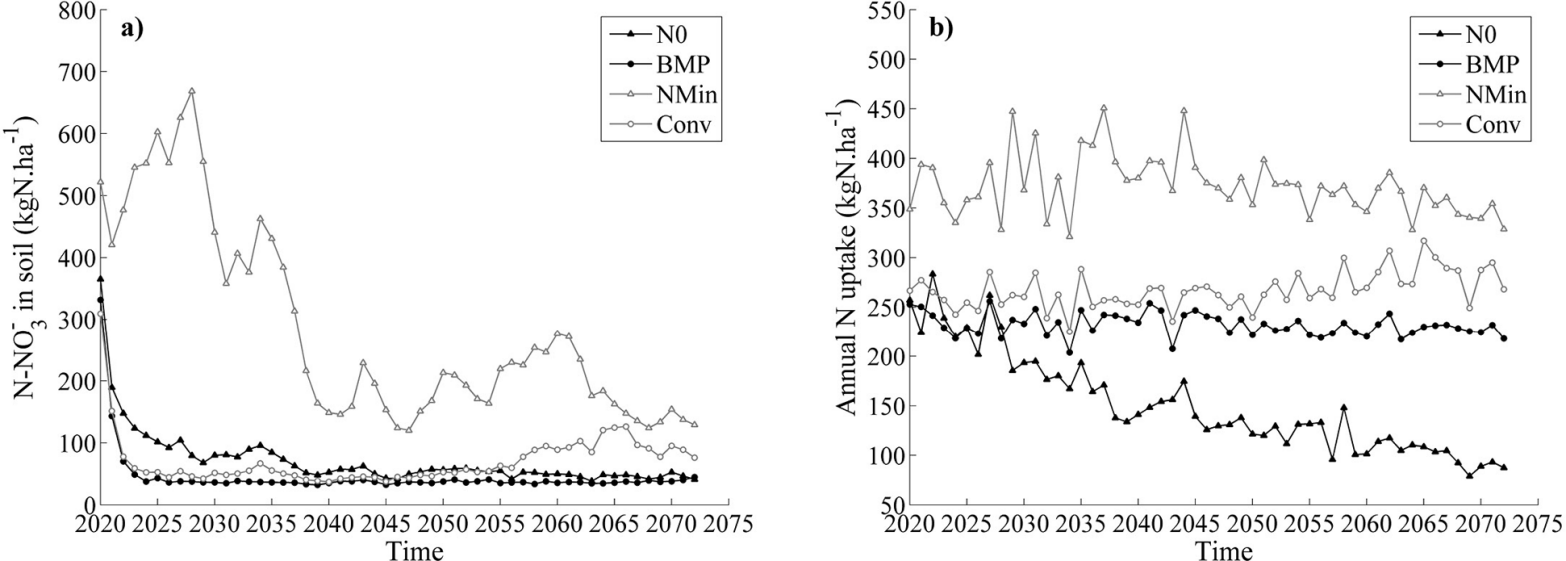
Simulated annual soil nitrate content and maize nitrogen uptake. Simulated median soil nitrate content (a) and annual median nitrogen uptake by the maize crop (b) for the NMIN (grey empty triangle line), CONV (grey empty circle line), N0 (black full triangle line) and BMP (black full circle line) treatments in the long term rotation (2020–2073).

Simulated soil NO_3_^-^ content (Fig 5A) for maize under future climate conditions was high for NMIN after the first year of the long-term simulations (521 kg N ha^-1^), but decreased by 75% over the simulated period, to reach 129 kg N ha^-1^ remaining in the soil. On the other hand, CONV treatment left 309 kg N ha^-1^ in the soil after the first year, and the simulation ended with 76 kg N ha^-1^ (75.4% reduction). Similarly, N remaining in the soil after the first year was also observed for BMP (331 kg N ha^-1^) and NO (365 kg N ha^-1^), while NO_3_^-^ was reduced in these treatments by 86–88% by the end of the simulated period. Simulated soil NO_3_^-^ contents showed similar trends for both emission scenarios.

Simulated maize N uptake showed different but consistent patterns for NMIN, CONV, and BMP but decreased sharply for the N0 treatment (Fig 5B). The average N uptake for NMIN, CONV and BMP equaled 372, 267 and 231 kg N ha^-1^ year^-1^ respectively, and were consistent between RCP scenarios (Fig 5B). Overall, the crop N uptake decreased to 3.2 kg N ha^-1^ year^-1^ for N0, both under RCP6.0 and RCP8.5.

**Fig 6:**
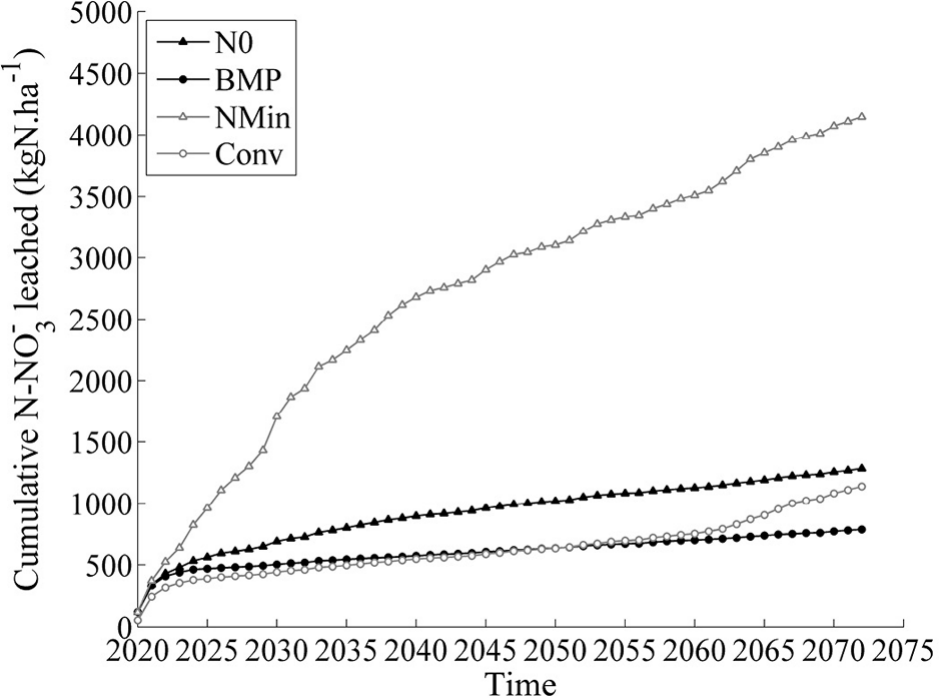
Simulated cumulative nitrate leaching. Simulated median cumulative nitrate leached for the NMIN (grey empty triangle line), CONV (grey empty circle line), N0 (black full triangle line) and BMP (black full circle line) treatments in the long term rotation (2020–2073) under RCP8.5.

**Fig 7:**
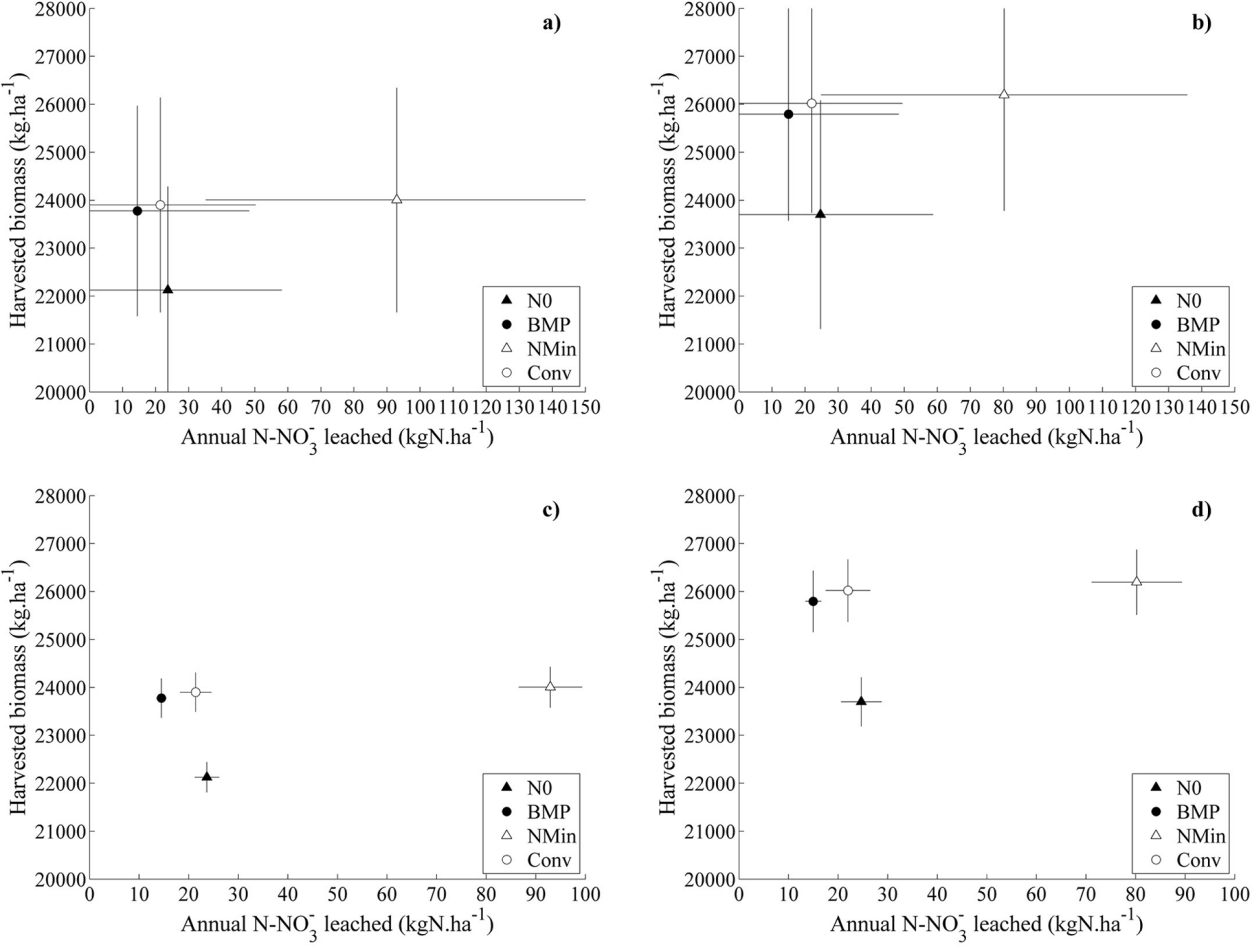
Inter-annual variability and inter-GCMs variability associated to nitrate leaching and harvested biomass. Average annual nitrate leached and harvest biomass for maize for the NMIN (open triangle), CONV (open circle), N0 (filled triangle) and BMP (filled circle) treatments in the long term rotation (2020–2073). Fig7a and b shows the inter-year variability when GCMs simulations are averaged. Fig7c and d shows the variability over the GCMs when average are computed over years. Fig7a and c refer to RCP6.0 scenario, while Fig7b and d refer to RCP8.5.

Simulated cumulative NO_3_^-^ leached increased over time for all the simulated treatments (Fig 6). Under N0 treatment, the cumulated NO_3_^-^ amount leached equaled 786 kg N ha^-1^ under RCP8.5 treatment (762 kg N ha^-1^ under RCP6.0). CONV management led to the leaching of 1139 and 1103 kg N ha^-1^ under RCP8.5 and RCP6.0 scenarios. NMIN management showed the highest losses, with 4150 and 4882 kg N ha^-1^ under RCP8.5 and RCP6.0 scenarios, respectively. The amount of NO_3_^-^ leached (median simulated values out the 29 GCMs) were thus systematically lower under RCP8.5 emission, compared to RCP6.0. The trade-off between maize biomass production and NO_3_^-^ leaching for the four simulated treatments can be seen in Fig 7. N0 produced the lowest biomass (23,780 kg ha^-1^ under RCP8.5) and was characterized by an average annual NO_3_^-^ leached of 23.9 kg N ha^-1^ year^-1^ (under RCP8.5). NMIN exhibited the highest simulated biomass yields (24,060–26,300 kg ha^-1^ on average, according to RCP6.0 and RCP8.5), but at the price of the highest leaching levels (92–78 kg N ha^-1^ year^-1^ under RCP6.0 and RCP8.5).

The BMP treatment resulted in one of the highest biomass values (23,830–25,905 kg ha^-1^) and the lowest annual NO_3_^-^ leaching (14.4–14.8 kg NO_3_^-^ ha^-1^ year^-1^), which highlights the importance of optimal use of Nitrogen for plant growth. Overall, CONV showed similar results to BMP in terms of biomass. Fig 7A and 7B show the inter-year variability when GCMs simulations are averaged. Fig 7C and 7D show the variability over the GCMs when averages are computed over the years. Wilcoxon tests were performed to compare the simulations averaged over GCM’s or years.

When considering inter-year variability (Fig 7A and 7B), no significant differences were found in biomass production between the fertilised treatments (NMIN, CONV and BMP), even though NO_3_^-^ leaching under BMP treatment was found to be highly significantly different (p-value < 0.001) from all other treatments.

When considering yield variability over the 29 GCMs (Fig 7C and 7D), BMP was statistically different (p-value < 0.01) from NMIN but not from CONV, while NMIN and CONV had biomass production values that were not significantly different. Statistical tests revealed the same results under both RCP 6.0 and RCP8.5 emission scenarios.

## Discussions

The application of N in the form of mineral fertilizers or organic [55, 56] amendments is necessary to achieve adequate levels of crop production and quality. However, this is often associated with significant environmental impacts due to the difficulty of matching crop N demand with N supply [5, 55–58].

SALUS was able to effectively reproduce the measured patterns of soil NO_3_^-^ and maize silage yields observed in data collected from an experiment over the course of two years. This suggests that the model could be used to test alternative N management strategies to abate NO_3_^-^ leaching and maintain crop biomass production under future climate conditions.

This study showed that agronomic practices aimed at minimizing NO_3_^-^ leaching under current conditions in NVZ [59] will not be sufficient to optimize the same economic and environmental benefits under future climatic conditions. BMP, as determined from data collected from two growing seasons, was the practice that best minimized leaching and maximized biomass production over the long term.

This study highlighted the challenge that current N management practices adopted to comply with the Directive 91/676 [41, 59], such as CONV and NMIN, will not perform well under projected climate change. Even though NO_3_^-^ concentrations in these treatments were slightly lower than the maximum threshold of 50 mg L^-1^ in Directive 91/676 (Table 6), simulations showed that the NUE of these management practices will be far from optimal under projected future conditions. Climate patterns predicted for this region are expected to bring higher temperatures from February to August and increased rainfall from August to October and in March (Fig 1C). During the summer months these environmental conditions increase the potential for crop stress which may result in a reduction of crop N uptake. In addition, projected changes in precipitation during the spring and autumn months increase the potential for NO_3_^-^ leaching.

In terms of crop N uptake, there were no important differences between CONV and BMP (30 kg N ha^-1^, Fig 5B**)**, which suggests that any effect of climate change will not be reflected in the crop’s ability to utilize soil N. However, substantial differences were noticed in soil NO_3_^-^ concentrations and the amount of NO_3_^-^ leached from the soil (Figs 5A and 6) among the different treatments. The reason for this result is twofold: first, the N application rate in maize under the BMP treatment (223 kg N ha^-1^) was slightly lower than under NMIN (230 kg N ha^-1^) and substantially lower than under CONV (304 kg N ha^-1^). Moreover, the separate application of liquid manure before sowing (173 kg N ha^-1^) and the in-season application of urea (50 kg N ha^-1^) maintained a high degree of synchronicity between plant N demand and N supply. As a result, N accumulation in the soil was limited and resulted in substantially lower nitrate leaching rates under both RCP6.0 and RCP8.5 scenarios (Figs 5A, 6 and 7). Accordingly, the NMIN and CONV systems showed the highest values of NO_3_^-^ leaching under projected changes in climate (Fig 5).

The reduced N rates of BMP also resulted in average plant N uptake levels (230 kg N ha^-1^) that were comparable to CONV (260 kg N ha^-1^) or substantially lower than in NMIN (360 kg N ha^-1^) as shown in Fig 5B and Table 9. Importantly, even though the N rate in BMP was reduced by 50kg N ha^-1^ compared to CONV and NMIN, crop N availability in BMP did not affect average biomass production (25 t dry matter ha^-1^), which was comparable to both NMIN and CONV under the RCP6.0 and RCP8.5 scenarios (Fig 7, Table 9). These results confirm the observations of [60] who reported no differences in maize biomass production between treatments fertilised with mineral N or with slurry in a Mediterranean environment.

Critically, the ratio between harvested biomass and N lost via leaching simulated for the BMP practice were substantially higher compared to the other treatments (Fig 7, Table 9).

A N management practice that is able to deliver high crop productivity and limit NO_3_^-^ leaching will be critical for future Mediterranean farming systems as climate change is projected to increase summer temperatures and autumn rainfall events. Accordingly, N management practices that will enable farmers to reduce N inefficiencies under these circumstances will result in substantial economic gains [5].

These results are critical for future agricultural practices in Mediterranean nitrate-vulnerable zones. Crop yields in these systems will need to be maintained and N leaching minimized even as changes in climate cause substantial shifts in temperature and rainfall patterns [61]. This study also highlights the potential for using crop models to predict crop response to different N management strategies and environmental stresses under future climatic scenarios. This methodology made it possible to identify BMP as the N management strategy best suited to comply with European regulations, since BMP demonstrates the ability to achieve high maize biomass production levels and minimize NO_3_^-^ leaching losses through the use of lower N inputs. Overall, the results of this research can assist farmers and policy makers to define the N management practices best suited to comply with Directive 91/676 [41] and maintain high crop productivity levels under changing climatic conditions.

## Conclusions

The scenarios simulated in this study illustrate the implications that future climate changes could have on N dynamics in a Mediterranean NVZ. While these results are influenced by the particular crops and soil characteristics of the site chosen, they provide insight into the potential to increase NUE and decrease nitrate leaching in cereal-based cropping systems in a Mediterranean NVZ.

Simulations from the SALUS model show that it reproduces the patterns of soil NO_3_^-^ and silage yield observed in field conditions. The model was used, along with future climate scenarios, to extrapolate the results an experiment conducted over two year into the future.

In the projected scenarios, the three N treatments assessed in the field study (N0, NMIN, CONV) were compared to a best management practice (BMP) chosen on the basis of observed crop N uptake from data collected during the two years of field trials. NMIN, CONV, and BMP showed similar crop N uptake over the long-term simulation (Fig 5B). However, NMIN and CONV showed higher NO_3_^-^ leaching than BMP. Therefore, the trade-off between the amount of biomass produced and the amount of NO_3_^-^ leached suggests that BMP was the best practice for reducing environmental pollution and maximizing production.

The SALUS crop model demonstrated its ability to reproduce field experiment results in the short term and its utility in projecting alternative management scenarios beyond a few years of experimental data into the future. Current conventional practices intended to minimize N loss will be inadequate in the future (Fig 6) because of changes in weather patterns. Importantly, farmers will be able to achieve substantial reductions in nitrate leaching by decreasing current mineral and organic fertilizer N rates without suffering yield penalties.

The results of this research will assist farmers and policy makers to define N management practices best suited to comply with the Directive 91/676 [41] while maintaining high productivity levels.
